# Discovery of Novel Small-Molecule Compounds with Selective Cytotoxicity for Burkitt’s Lymphoma Cells Using 3D Ligand-Based Virtual Screening

**DOI:** 10.3390/molecules191119209

**Published:** 2014-11-19

**Authors:** Martina Gobec, Izidor Sosič, Boris Brus, Aleš Obreza, Stanislav Gobec, Irena Mlinarič-Raščan

**Affiliations:** University of Ljubljana, Faculty of Pharmacy, Aškerčeva cesta 7, Ljubljana 1000, Slovenia; E-Mails: martina.gobec@ffa.uni-lj.si (M.G.); izidor.sosic@ffa.uni-lj.si (I.S.); boris.brus@ffa.uni-lj.si (B.B.); ales.obreza@ffa.uni-lj.si (A.O.)

**Keywords:** Burkitt’s lymphoma, ligand-based, similarity search, selectivity

## Abstract

We describe a ligand-based approach towards compounds with more specific targeting for Burkitt’s lymphoma. Using three-dimensional ligand-based similarity searches and a previously described hit compound, we have identified six compounds that are chemically different but with similar spatial conformations. Biological evaluation revealed that one compound has better growth inhibition and improved selectivity towards Burkitt’s lymphoma cells than the query compound. However, initial mechanism-of-action studies show a different target profile in comparison with the previous hit compound, which does not involve the inhibition of the proteasome or the NFκB pathway. The data from this study provide a solid basis for further efforts in the search for selective agents against Burkitt’s lymphoma.

## 1. Introduction

Burkitt’s lymphoma is considered to be one of the clinically most aggressive of B-cell lymphomas. It is characterized by a translocation of the proto-oncogene *c-Myc* and immunoglobulin promoter regions, which leads to constitutive expression of the MYC protein [1,2]. Burkitt’s lymphoma most commonly affects children (representing 30%–50% of childhood non-Hodgkin’s lymphomas) and young adults. If left untreated, this disease is rapidly fatal, as it progresses extremely fast (doubling time, 24–48 h), and it has a high propensity to metastasize into the central nervous system [3]. Therapeutic intervention is initiated immediately, as it is accepted that the high growth fraction and the short doubling time of Burkitt’s lymphoma make intensive short-cycle chemotherapy a necessity. Treatments usually combine etoposide, prednisone, vincristine, cyclophosphamide, doxorubicine and rituximab (e.g., the EPOCH-R regiment) [4,5,6]. Due to the intense chemotherapy, there are often acute cytotoxic effects, including neutropenia, thrombocytopenia, myelosuppression, nausea and vomiting, neurotoxicity, hyperglycemia, stomach problems, cardiotoxicity, hair loss, constipation, and hypotension [3,5,7].

Achieving selective cytotoxicity against malignantly transformed cells remains the main goal in the development of anticancer therapeutics. Therefore, a continuous search for novel, more target-specific agents is needed. Using this approach, the switch of equilibrium between tumor-cell progression and cell death in favor of disease regression is possible, while sparing normal tissue. In our previous study, we demonstrated that compounds with the *N*-amidinopiperidine moiety are selectively cytotoxic towards malignantly transformed B-cells (Ramos and Daudi Burkitt’s lymphoma cells). The molecular pathways involve disruption of NFκB through inhibition of the trypsin-like activity of the proteasome [8].

**Figure 1:**
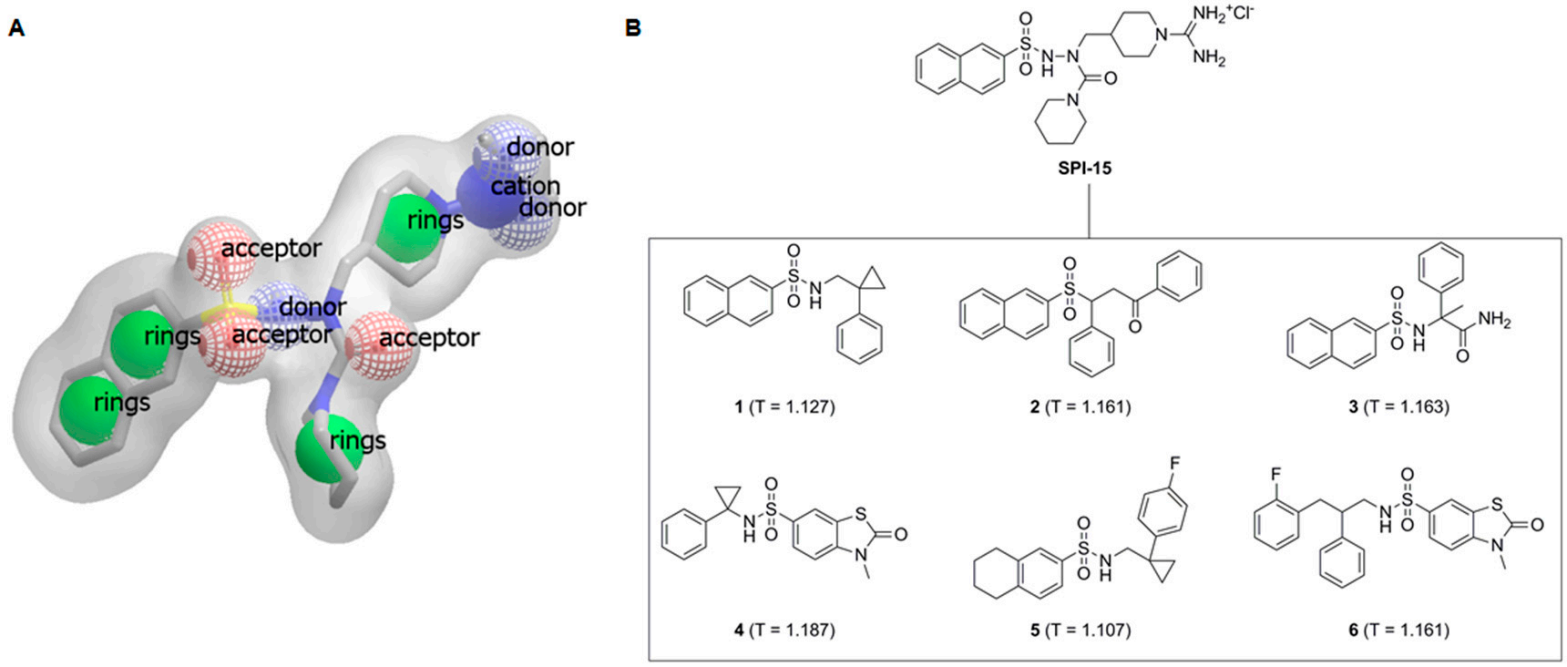
(**A**) Shape and features of compound SPI-15, as perceived by the rapid overlay of chemical structures (ROCS) software; (**B**) Structural formulae of **SPI-15** and its spatial analogs investigated here with their TanimotoCombo scores, Compounds **1**–**6**, T = TanimotoCombo score.

The overall objective of the present study was a search for improved inducers of apoptosis that show enhanced toxicity towards B-cell malignancies. To achieve this goal, a ligand-based three-dimensional (3D) similarity search was initiated, based on a previously described selective compound **SPI-15** (Figure 1). We used the method of rapid overlay of chemical structures (ROCS), which performs shape-based overlays of conformers of a candidate molecule to a query molecule, in one or more conformations. The algorithm is based on the shape comparison and overlap of the volume of two molecules. In comparison to 2D similarity search methods, the new lead structures retrieved usually show relatively diverse chemical structures, and therefore enable scaffold hopping to be performed, and chemical diversity of a given set of compounds to be acquired [9,10].

## 2. Results and Discussion

### 2.1. Identification and Evaluation of Three-Dimensional Spatial Analogs

Our previous investigations identified *N*-amidinopiperidine-based compounds that show selective cytotoxicity towards Burkitt’s lymphoma cells (e.g., Ramos cells) [8]. Of these compounds, **SPI-15** (Figure 1) showed the lowest IC_50_ (18 µM). With the aim to identify novel compounds with enhanced toxicities towards B-cell malignancies and improved pharmacodynamics and physicochemical properties, we initially conducted a ligand-based 3D similarity search.

Direct comparisons of virtual screening data of several structure-based virtual screening campaigns revealed that a ligand-based ranking method (e.g., ROCS) performs at least as well as, and often even better than, docking methods [11]. In the experiments described here, **SPI-15** was used as the query molecule. ROCS 3.1.1 was used to compare the compounds in the ZINC drug-like database with all query conformers [11]. The single best overlay hits were ranked according to the TanimotoCombo scoring function, which considers similarities in the molecular shape and color (*i.e.*, atom types). The top six ranked compounds (highest similarity to **SPI-15**) that were commercially available were purchased (Figure 1).

**Figure 2:**
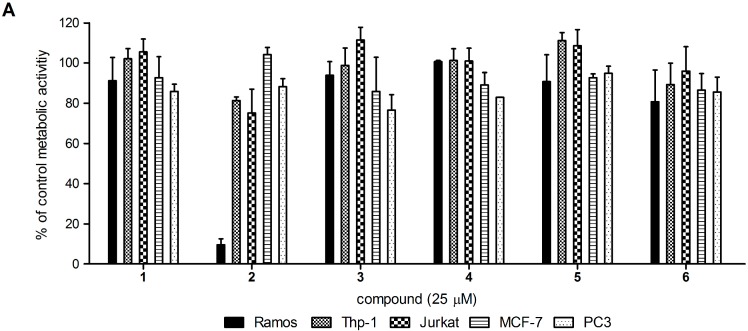

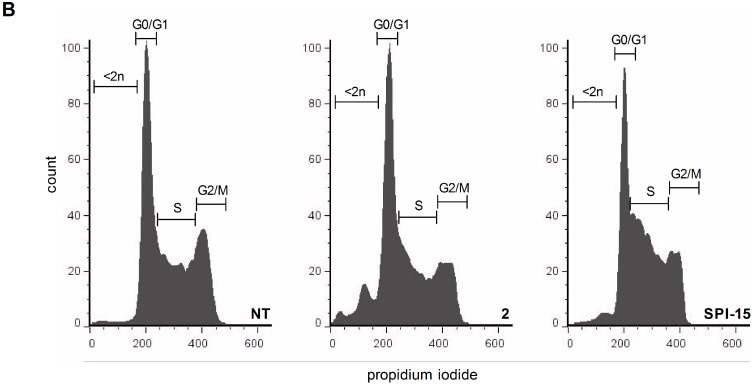
(**A**) Ramos, Thp-1, Jurkat, MCF-7, and PC3 cells were incubated with vehicle (control) and 25 µM Compounds **1**–**6** for 24 h. The data are the residual metabolic activities expressed as percentages relative to the control cells, as means (±SD) from three independent experiments, each conducted in triplicate; (**B**) Cell cycle analysis of Ramos cells after 24 h treatment with vehicle (NT), Compound **2** and **SPI-15** (as indicated). The data are representative of a single experiment, of three independent experiments.

Next, induction of cell death by these 3D spatial analogs, Compounds **1**–**6**, was investigated. Using a metabolic activity assay (3-[4,5-dimethylthiazol-2-yl]-5-[3-carboxymethoxyphenyl]-2-[4-sulfophenyl]-2H-tetrazolium; MTS), we evaluated the proliferation rates of cells representative of several cancer types: Ramos (Burkitt’s lymphoma), Jurkat (human T-cell leukemia), Thp-1 (acute monocytic leukemia), MCF-7 (breast cancer) and PC3 (prostate cancer) cells. The cells were treated for 24 h with the relevant vehicle and the compounds of interest at 25 µM (higher concentrations were not used due to the poor solubilities of Compounds **1**–**6** in cell-culture media). Comparisons of these metabolic activities revealed that only Compound **2** was selectively cytotoxic for Ramos cells, while the other 3D analogs had little or no effects on the viabilities of any of the cell types assayed (Figure 2A). For Compound **2**, the IC_50_ for Ramos cells was 6.7 µM, which is almost a third of that for **SPI-15** (18 µM) [8].

To determine whether this decrease in metabolic activity was due to increased cell death, cell cycle analysis was performed. Ramos cells were treated for 24 h with 10 µM Compound **2** or 25 µM **SPI-15**, as this concentration of **SPI-15** was previously shown to arrest Ramos cells in S phase [8]. Here, comparable data were obtained for **SPI-15**, while Compound **2** had no effects on the cell-cycle distribution when compared with the control. However, an increase in Ramos cells with hypodiploid DNA content was observed following the treatment with Compound **2** (from 3% to 12%), which indicated that the decreased metabolic activity was due to increased cell death (Figure 2B).

### 2.2. Induction of Caspase-Dependent Apoptosis

**Figure 3:**
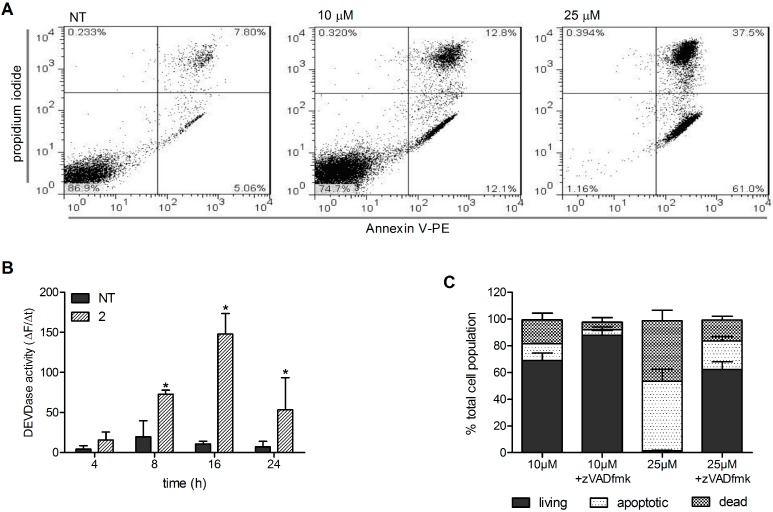
(**A**) Determination of annexin-V-positive and propidium-iodide-positive Ramos cells after treatment with vehicle (NT) and 10 µM and 25 µM Compound **2** (as indicated) for 24 h, with analysis by flow cytometry. The data are representative of a single experiment, of three independent experiments; (**B**) Ramos cells were treated for 4, 8, 16, and 24 h with vehicle (NT) and 25 µM Compound **2**. Caspase 3/7 activity was determined spectrofluorometrically by measuring cleavage of Ac-DEVD.AFC in whole-cell lysates. The data are changes in fluorescence with time, as means (±SD) from three independent experiments, each conducted in triplicate. *, *p* < 0.05, *versus* relevant vehicle control; (**C**) Analysis of annexin-V/propidium-iodide–positive cells after 24 h treatment with 10 µM and 25 µM Compound **2** in the absence and presence of 50 µM zVADfmk. The data are means (±SD) from three independent experiments, each conducted in triplicate.

The mechanism of Ramos cell death induced by Compound **2** was further investigated. These cells were treated with 5 µM, 10 µM, and 25 µM Compound **2** for 24 h. Then, the externalisation of phosphatidylserine (a hallmark of apoptotic cell death) was determined using the annexin V-FITC/propidium iodide assay. As shown in Figure 3, Compound **2** induced a concentration-dependent increase in apoptotic (*i.e.*, annexin-V-positive) and dead (annexin-V-positive and propidium-iodide-positive) cells (Figure 3A). Induction of apoptosis was additionally defined with the detection of caspase 3/7 activity that initially increased with time, then peaked at 16 h, and subsequently decreased (Figure 3B).

To confirm that Compound **2** induces caspase-dependent cell death, Ramos cells were pretreated in the absence and presence of 50 µM zVad-fmk (a pan-caspase inhibitor) for 1 h. Then, 10 µM or 25 µM Compound **2** was added, and the cells were incubated for a further 24 h. In the presence of zVad-fmk there was a significant decrease in Compound-**2**–induced apoptotic and dead cells (Figure 3C), which indicated that these apoptotic processes are caspase dependent. Taken together, these data show that Compound **2** caused caspase-dependent apoptosis of Ramos cells.

### 2.3. Molecular Mechanisms of Cytotoxicity Do Not Involve the NFκB Pathway

**Figure 4:**
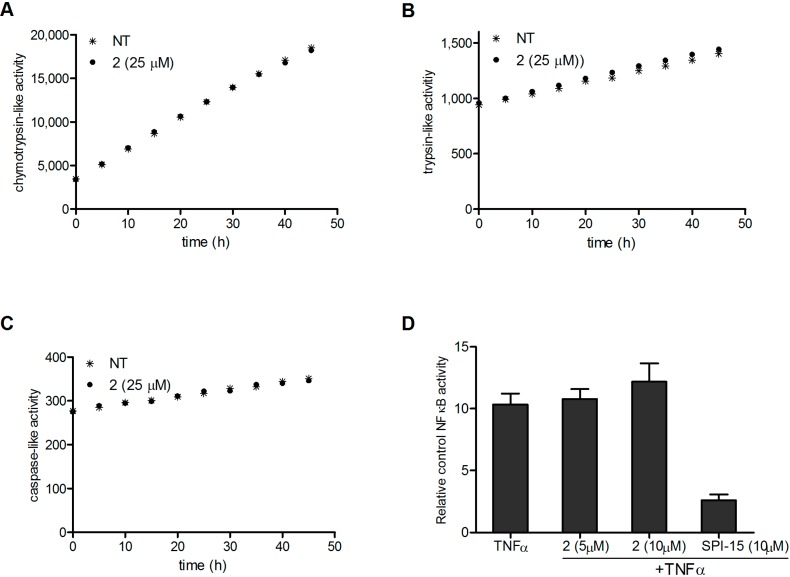
The 20S human proteasome activities were determined spectrofluorometrically according to the cleavage of the substrate. The chymotrypsin-like (**A**); trypsin-like (**B**); and caspase-like (**C**); (**D**) Determination of SEAP activity in Ramos-blue cells after pre-treatment for 1 h with vehicle, 5 µM and 10 µM Compound 2 or 10 µM SPI-15, and subsequently adding 50 ng/mL TNFα. After 16 h, the supernatants were collected and the SEAP activities were determined. The data are means (±SD) from three independent experiments, each conducted in triplicate.

Compound **SPI-15** was previously shown to inhibit NFκB through inhibition of proteasomal activity. As Compound **2** was identified as a spatial analog of **SPI-15**, we hypothesised that the molecular mechanisms underlying the induction of apoptosis involve modulation of similar targets. Therefore, we next determined whether Compound **2** can inhibit the proteasome. Inhibition of the activities of all three proteasomal subunits (*i.e.*, chymotrypsin-like, trypsin-like, caspase-like) by Compound **2** was determined. Figure 4 shows that 25 µM Compound **2** had no effects on the proteasomal activities of any of these catalytically active subunits.

Although the proteasome was not affected by Compound **2**, modulation of the NFκB signaling pathway was not excluded. As constitutive NFκB activation is a common feature of most hematological malignancies, and as this is believed to be crucial for the survival of these malignant cells [12], we next evaluated the potential modulation of the NFκB pathway by Compound **2**. The Ramos-Blue reporter cell line was used here, which stably expresses an NF-κB/AP-1–inducible SEAP reporter construct, to allow detection of the modulation of the NFκB pathway. These cells were pre-treated with at 5 µM and 10 µM Compound **2** or 10 µM **SPI-15** (higher concentrations were not used due to the toxicity) for 1 h, and then they were stimulated with a known NFκB–activating stimulus, 50 ng/mL recombinant TNF-α. Determination of the SEAP activity in the supernatant after 16 h demonstrated that Compound **2** had no effects on TNF-α–induced activation of NFκB, while the control (**SPI-15**) caused an over 4-time decrease of the activity. This indicates that the apoptosis induced by Compound **2** does not involve the NFκB signaling pathway. In conclusion, Compound **2** appears to act through other molecular mechanisms, and to have a different target profile in comparison with the query compound, **SPI-15**.

## 3. Experimental Section

### 3.1. Computer Hardware and Compound Database Preparation

The computational study was carried out on a workstation with four dual-core AMD Opteron 2.0-GHz processors, 16 GB RAM, four 320-GB hard drives in a RAID10 array, and Nvidia GeForce 7900 graphic cards, running 64-bit Fedora 7. For the virtual screening, the ZINC drug-like subset was used, which now includes 11.3 million drug-like compounds [13]. To cover as much conformational space as possible, the database was first processed with the Omega 2.4.3 software (OpenEye Scientific Software Inc, Santa Fe, NM, USA) using the default settings, which yielded an average of 152 conformations per compound [14,15].

### 3.2. Ligand-Based Virtual Screening

For the ligand-based 3D similarity search, the compound **SPI-15** was used as a query molecule [8]. The Omega 2.4.3 software was used to generate six conformers of **SPI-15** (by the default settings the RMS values between the conformers are 0.5 Å or more). To compare the molecules in the ZINC drug-like database to all of the query conformers, ROCS 3.1.1 (OpenEye Scientific Software Inc., Santa Fe, NM, USA) was used, with the default settings [11]. The single best overlay hits were ranked according to the TanimotoCombo scoring function, which considers similarities in the molecular shapes and colors (*i.e.*, atom types). From the compounds with the highest similarities to **SPI-15**, six that were available were evaluated *in vitro* (Figure 1B, Compounds **1**–**6**).

### 3.3. Materials and Methods

Compounds **1**–**6** were purchased from Asinex Chemicals (Winston-Salem, NC, USA). Prior to use in the biochemical assays, the purities of all of the compounds were determined by HPLC. Compound **2** was subsequently carefully characterized. The melting point of Compound **2** was determined on a Reichert hot-stage apparatus, and is uncorrected. The ^1^H-NMR spectrum was recorded on a Bruker Avance 400 DPX spectrometer (Bruker BioSpin Corporation, Billerica, MA, USA) at 295 K, and is reported as ppm, using solvent as an internal standard (DMSO-*d_6_* at 2.50 ppm). The coupling constants (*J*) are given in Hz, and the splitting patterns are designated as: s, singlet; d, doublet; dd, double doublet; and m, multiplet. The ^13^C-NMR spectrum was recorded on a Bruker Avance 400 spectrometer at 295 K, and is reported in ppm using solvent as internal standard. Infrared (IR) spectrum was recorded on Fourier transform-IR spectrometer (Thermo Nicolet Nexus 470 ESP, Thermo Fisher Scietific Inc, Waltham, MA, USA). Mass spectrometry and high-resolution mass measurement was performed on a VG-Analytical Autospec Q mass spectrometer (VG Analytical, Manchester, UK). The HPLC analysis was run on an Agilent 1100 system equipped with a quaternary pump and a multiple wavelength detector. An Agilent Eclipse C18 column (4.6 mm × 50 mm; 5 mm) was used, with a flow rate of 1.0 mL/min, detection at 254 nm, and an eluent system of: A, 0.1% *trifluoroacetic acid* in H_2_O; B, MeOH. The following gradient was applied: 0–3 min, 40% B; 3–18 min, 40%–80% B; 18–23 min, 80% B; 23–30 min, 80%–40% B; run time, 30 min; temperature, 25 °C.

#### *3-(Naphthalen-2-ylsulfonyl)-1,3-diphenylpropan-1-one* (**2**) (Scheme 1)

**Scheme 1:**
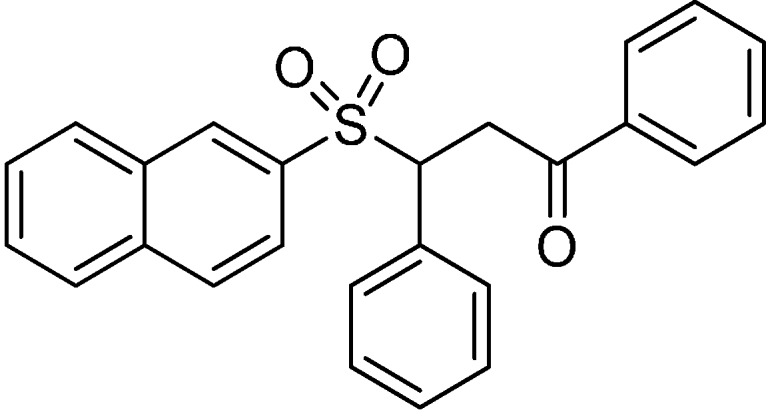
Structural formula of hit compound **2**.

White solid. Mp = 179.0–181.0 °C; ^1^H-NMR (400 MHz, DMSO-*d_6_*) δ (ppm) = 3.97 (dd, B part of ABX, *J*_AB_ = 18.0 Hz, *J*_BX_ = 4.5 Hz, COCH_2_CH, 1H), 4.10 (dd, A part of ABX, *J*_AB_ = 18.0 Hz, *J*_AX_ = 9.0 Hz, COCH_2_CH, 1H), 5.19 (dd, X part of ABX, *J*_AX_ = 9.0 Hz, *J*_BX_ = 4.5 Hz, COCH_2_CH, 1H), 7.18–7.27 (m, 3H, Ar-H), 7.29–7.33 (m, 2H, Ar-H), 7.46–7.51 (m, 2H, Ar-H), 7.60–7.68 (m, 3H, Ar-H), 7.70–7.75 (m, 1H, Ar-H), 7.92–7.96 (m, 2H, Ar-H), 8.02–8.10 (m, 3H, Ar-H), 8.35 (d, *J* = 1.5 Hz, 1H, Ar-H); ^13^C-NMR (100 MHz, DMSO-*d_6_*) δ (ppm) = 36.70, 65.41, 123.35, 127.58, 127.77, 128.06 (2C), 128.51, 128.66, 128.92, 129.37, 129.40, 129.97, 130.57, 131.43, 132.31, 133.57, 133.92, 134.69, 135.82, 195.13; IR (ATR) υ = 3406, 3052, 1681, 1593, 1448, 1338, 1303, 1233, 1141, 1124, 1072, 982, 747, 684 cm^−1^; ESI-MS [M+H]^+^: *m/z* = 401; HRMS (ESI) m/z calculated for C_25_H_21_O_3_S [M+H]+ 401.0773, found 401.0768; purity by HPLC: 99.10%.

### 3.4. Cell Culture

The Ramos, Thp-1, Jurkat, PC3 and MCF-7 cell lines were from ATCC (LGC Standards, Middlesex, UK), and Ramos-Blue cells were from Invivogen (San Diego, CA, USA). The Ramos, Thp-1 and Jurkat cell lines were cultured in RPMI 1640 medium (Sigma-Aldrich, St. Louis, MO, USA) supplemented with 10% fetal bovine serum (Gibco, Grand Island, NY, USA), 2 mM L-glutamine, 100 U/mL penicillin, 100 µg/mL streptomycin and 50 µM 2-mercaptoethanol (all from Sigma-Aldrich), in a humidified chamber at 37 °C and 5% CO_2_. PC3 cells were cultured in advanced *Dulbecco’s Modified Eagle’s medium* (DMEM)/F-12 (1:1; Gibco, Grand Island, NY, USA), HOS cells were cultured in DMEM (Sigma-Aldrich), and MCF-7 cells were cultured in advanced DMEM/F-12 with 10 μg/mL insulin, 20 ng/mL epidermal growth factor, and 0.5 μg/mL hydrocortisone (all from Sigma-Aldrich). Each medium was supplemented with 10% fetal bovine serum (Gibco), 2 mM l-glutamine, 100 U/mL penicillin, and 100 µg/mL streptomycin. The Ramos-Blue cells were cultured according to the manufacturer instructions.

### 3.5. Metabolic Activity Assay

The cells (100 μL; 2 × 10^5^ cells/mL) were treated with the appropriate vehicle (control cells) and the various concentrations of the compounds of interest, in triplicate, in 96-well plates. The metabolic activities were assessed using the CellTiter 96^®^ Aqueous One Solution Cell Proliferation Assay (Promega, Fitchburg, WI, USA), according to the manufacturer instructions.

### 3.6. Cell-Cycle Analysis

The Ramos cells (5 × 10^5^ cells) were treated with the vehicle and the compounds of interest for 24 h. The cells were then washed with phosphate-buffered saline (PBS) and fixed with 80% ethanol at –20 °C for 15 min. The fixed cells were pelleted by centrifugation and rehydrated in 5 mL PBS for 15 min at room temperature. The collected cells were resuspended in 500 µL staining buffer (3 µM propidium iodide, 100 mM Tris, pH 7.4, 150 mM NaCl, 1 mM CaCl_2_, 0.5 mM MgCl_2_, 0.1% Nonident P-40). After a 15 min incubation, the samples were analyzed using a FACScalibur and evaluated using the FlowJo software (BD Bioscience, Franklin Lakes, NJ, USA).

### 3.7. Annexin V Assay

We used annexin V-FITC Apoptosis Detection kits (Sigma-Aldrich), according to the manufacturer instructions. Briefly, following treatments with vehicle and the compound of interest, the Ramos cells were washed with PBS and resuspended in kit’s binding buffer. The cell suspensions were transferred to 5 mL tubes and the annexin V and propidium iodide were added. The cell suspensions were gently vortexed and incubated for 15 min at room temperature in the dark, followed by analysis using a FACScalibur flow cytometer (BD Bioscience, Franklin Lakes, NJ, USA).

### 3.8. Determination of Caspase 3/7 Activity

Caspase 3/7 activity was measured in total cell lysates using the fluorescent *N*-Acetyl-Asp-Glu-Val-Asp-7-amido-4-trifluoromethylcoumarin (Ac-DEVD-AFC) substrate (Bachem, Bubendorf, Switzerland). The Ramos cells incubated with vehicle or compounds for the indicated times were washed twice in PBS and resuspended in 200 µL ice-cold caspase lysis buffer (0.1% Triton X-100, 100 mM phosphate buffer, pH 6.0, 1.3 mM EDTA, 100 mM NaCl), sonicated, and left on ice for 30 min. After centrifugation (14,000× *g*, 15 min, 4 °C), the total protein concentrations in the supernatants were measured using BioRad Protein Assay kits (Bio-Rad, Hercules, CA, USA), according to the manufacturer instructions. Cell lysates (20 μg protein) were incubated for 30 min at 37 °C in caspase reaction buffer (20 mM PIPES, pH 7.2, 10% sucrose, 0.1% CHAPS, 1 mM EDTA, 100 mM NaCl), after which 100 µM Ac-DEVD-AFC peptide substrate was added. Immediately following substrate addition, the fluorescence intensities were monitored continuously for 30 min using a fluorescence microplate reader (BioTek Synergy HT, BioTek, Winooski, VT, USA) at the excitation and emission wavelengths of 405 nm and 535 nm, respectively. Data are expressed as the relative increase in fluorescence as a function of time (ΔF/Δt).

### 3.9. Quanti-Blue Assay

Ramos-Blue cells stably expressing an NFκB/AP-1-inducible secreted embryonic alkaline phosphate (SEAP) reporter construct were assayed for NFκB transcriptional activity changes upon pretreatment with vehicle and the compounds of interest for 1 h, and subsequent stimulation with 50 ng/mL recombinant TNF-α. The SEAP activity was determined in the supernatants, according to the manufacturer instructions. Briefly, the cell supernatants (40 µL) were added to 160 µL QUANTI-Blue reagent and incubated at 37 °C for 2 h. The absorbance was measured on a microplate reader (BioTek Synergy HT), at 640 nm.

### 3.10. Proteasome Activity Measurements

Purified human 20S proteasome (1 μg/mL; Boston Biochem, Inc., Cambridge, MA, USA) was incubated with vehicle or the compounds of interest at 37 °C for 30 min in assay buffer (20 mM Tris, pH 8.0, 0.5 mM EDTA, 1 mM dithiothreitol). Then, 100 μM Suc-Leu-Leu-Val-Tyr-7-amino-4-methylcoumarin (AMC), Boc-Leu-Leu-Arg-Arg-AMC, and Z-Leu-Leu-Glu-AMC (Bachem, Bubendorf, Switzerland) were added for the assessment of the chymotrypsin-like, trypsin-like and caspase-like activities, respectively. The flourescence of the released AMC was continuously monitored at the excitation and emission wavelengths of 320 nm and 420 nm, respectively, at 37 °C, using an automated microplate reader (BioTek Synergy HT). The measured relative fluorescence (RFU) was monitored over time.

### 3.11. Statistics

All experiments were performed at least three times, with average values expressed as means ± SD. Statistical significance was determined by the Student’s *t*-test. Differences were considered significant for *p* < 0.05.

## 4. Conclusions

This computationally supported search for novel structural analogs of **SPI-15** resulted in the identification of six structurally related compounds. The initial screening showed a comparable toxicity profile to **SPI-15** for one of these spatial analogs, Compound **2**. Indeed, as well as having lower molecular mass and better ligand efficiency, Compound **2** showed improved activity, as a lower concentration was needed to induce caspase-dependent apoptosis of Ramos Burkitt’s lymphoma cells, in comparison with the parent compound, **SPI-15**. However, the molecular mechanisms of Compound **2** that lead to cell death are distinct to those of **SPI-15**, as Compound **2** does not involve inhibition of the proteasome or the NFκB pathway.

Further studies are needed to elucidate the molecular mechanisms and/or target(s) that are modulated by Compound **2**. Despite significant differences in the molecular mechanisms, we believe that the lower concentrations needed to have the desirable effects represent a promising step towards improved and more selective agents against Burkitt’s lymphoma. In addition, the data presented here provide a solid basis for further ligand-based design of improved compounds, and more importantly, for the identification of the target(s) involved in Burkitt’s lymphoma. This target identification will undoubtedly provide valuable information for future structure-based optimization efforts in the battle against this disease, and against other hematological malignancies.
